# Supplementary figures and images for: TAL Effectors with Avirulence Activity in African Strains of Xanthomonas oryzae pv. oryzae
Source: Rice (N Y). 2022 Feb 4;15:9. doi: 10.1186/s12284-022-00553-9 (PMC8816977; doi:10.1186/s12284-022-00553-9)

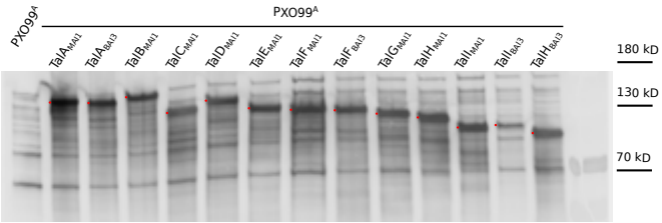

Supplement: Supplementary file 1 — Additional file 1: Fig. S1. Western-blot of PXO99A Xoo transformant total protein extracts using an anti-TALE antibody. Protein extracts prepared from the wild-type Xoo strain PXO99A and the Xoo strain PXO99A carrying, on the vector pSKX1, each of the nine tal genes of the Malian strain MAI1 and their variants in the Burkinabe strain BAI3. Reference molecular weights are indicated at right. The expressed MAI1 and BAI3 TALE proteins are each indicated by a red dot. Bands of lower molecular weight not present in the wild-type PXO99A extract are degradation products of the heterologously expressed TALEs, commonly observed. [file 12284_2022_553_MOESM1_ESM.pdf]

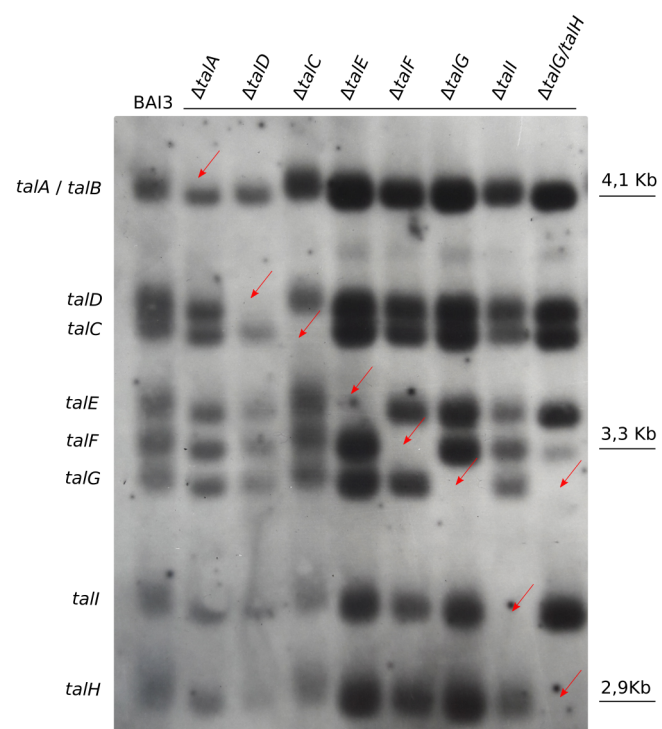

Supplement: Supplementary file 2 — Additional file 2: Fig. S2. Molecular characterization of a library of BAI3∆tal mutant strains. Genomic DNA of the wild-type Xoo strain BAI3 and derivative BAI3∆tal mutants were digested by BamHI-HF which cuts on either side of the central repeat region of tal genes and revealed by Southern blot using a 725-bp C-terminal talCMAI1 amplicon as probe (Yu et al. 2011). Individual mutants were obtained for each tal gene with the exception of talH. One double talG/talH mutant was analyzed instead. BAI3ΔtalB was obtained previously, also using the suicide plasmid pSM7 (Tran et al., 2018), and is therefore not included here. tal genes are indicated to the left and DNA sizes to the right. Red arrows indicate the tal gene(s) that were mutated. [file 12284_2022_553_MOESM2_ESM.pdf]

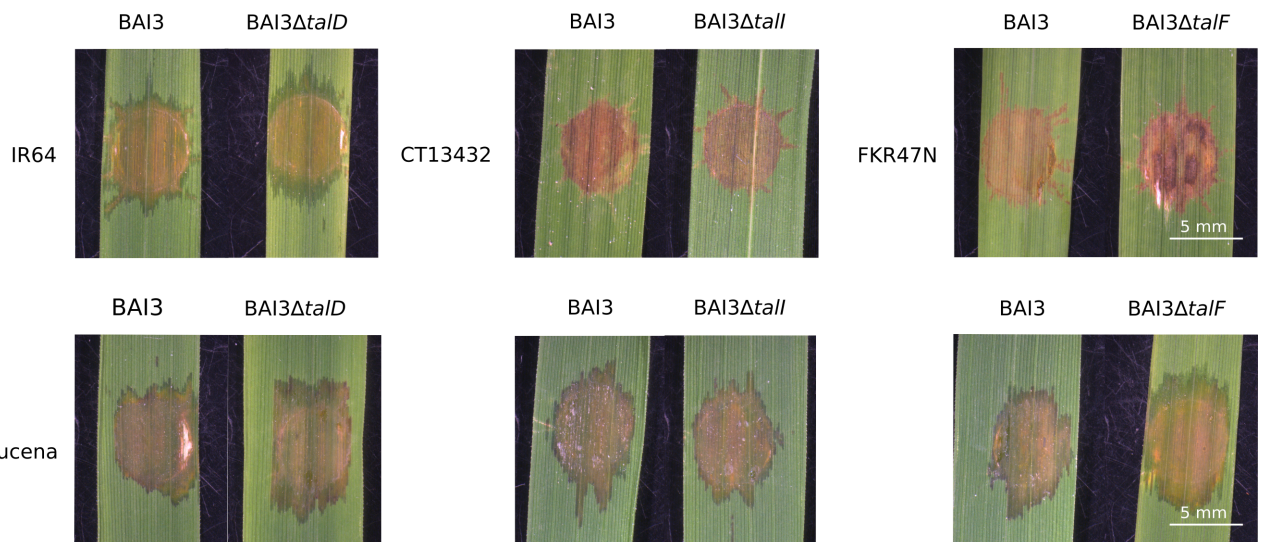

Supplement: Supplementary file 3 — Additional file 3: Fig. S3. Phenotypic responses upon leaf-infiltration of CT13432 and FKR47N plants with BAI3∆talI and BAI3∆talF mutants. Leaves of rice varieties IR64, CT13432, FKR47N and Azucena were infiltrated with the wild type African Xoo strain BAI3 and the mutant derivatives BAI3ΔtalD, BAI3ΔtalI and BAI3ΔtalF. Inoculated leaves were photographed at 5 dpi. [file 12284_2022_553_MOESM3_ESM.pdf]

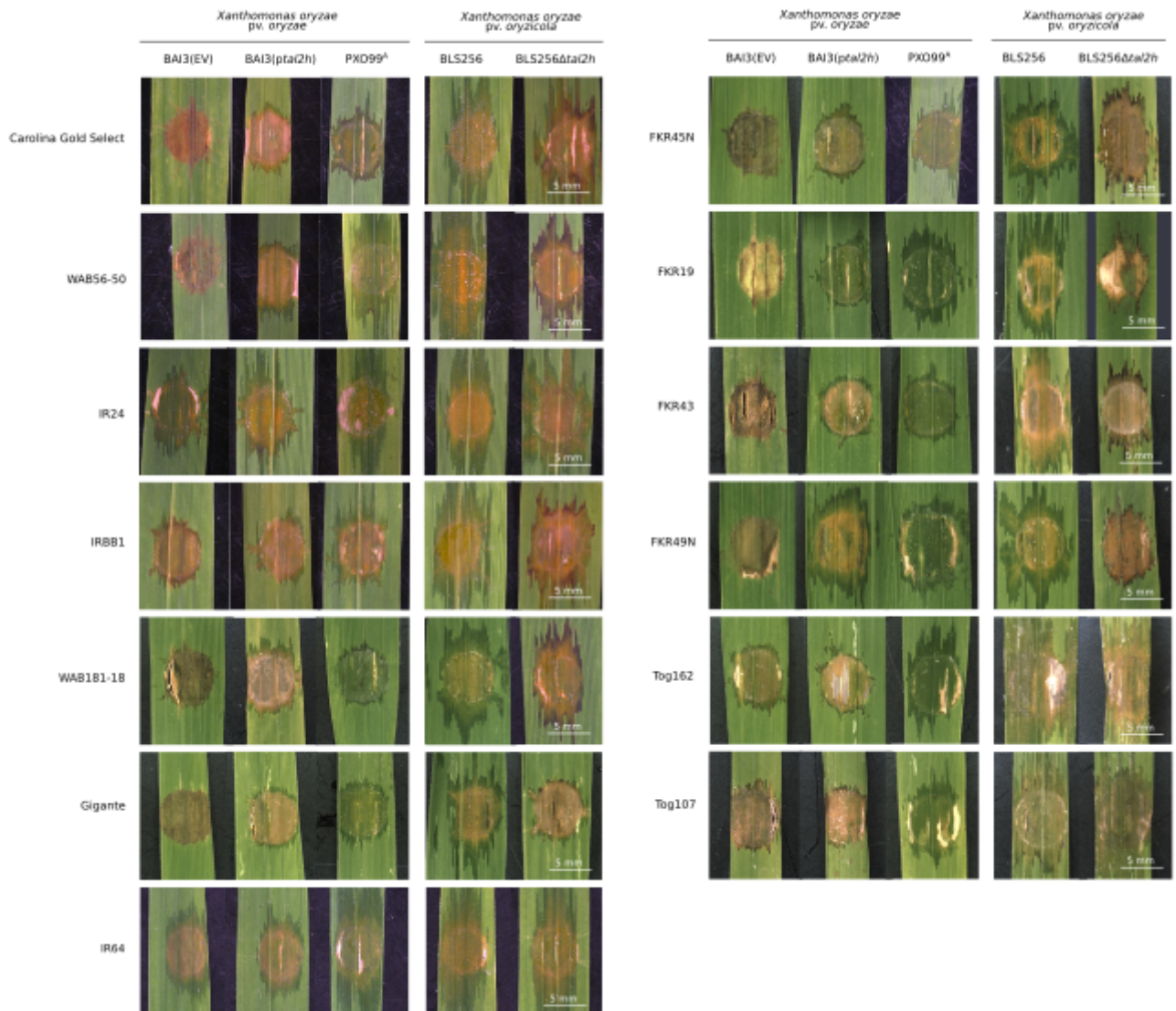

Supplement: Supplementary file 4 — Additional file 4: Fig. S4. The Tal2h truncTALE reveals Xa1-like resistance against the Xoo strain BAI3 in several rice varieties. Leaves of rice accessions, including Carolina Gold Select which carries Xo1, and IRBB1 which carries Xa1, were infiltrated with Xoc strain BLS256 which naturally carries the tal2h truncTALE gene, the mutant strain BLS256Δtal2h, as well as with Xoo strain BAI3 carrying an empty vector (EV) or tal2h. The Asian Xoo strain PXO99A which harbors two truncTALEs, was used as an additional positive control. Leaves were photographed at 5 dpi. [file 12284_2022_553_MOESM4_ESM.pdf]
