# Supplementary material for: TAL Effectors with Avirulence Activity in African Strains of Xanthomonas oryzae pv. oryzae
Source: Rice (N Y). 2022 Feb 4;15:9. doi: 10.1186/s12284-022-00553-9 (PMC8816977; doi:10.1186/s12284-022-00553-9)
Supplement: Supplementary file 5 — Additional file 5: Fig. S5. Western-blot analysis of Xoo total protein extracts using an anti-TALE antibody. Proteins extracts prepared from Xoo strain BAI3 carrying the empty vector pKEB31 (EV), BAI3ΔtalI carrying the empty vector pKEB31 (EV), BAI3 carrying the tal2h truncTALE gene, and BAI3ΔtalI with tal2h and the pSKX1 empty vector (EV), or pSKX1 containing talIBAI3 or talIMAI1 or talIMAI1ΔAD. BAI3 tal genes and tal2h are indicated to the left and right, respectively. Molecular weight is indicated. [file 12284_2022_553_MOESM5_ESM.pdf]

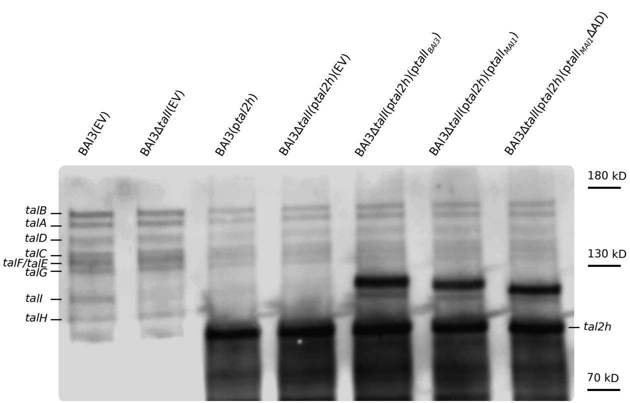

BAI3(EV)
BAI3Δ*talI*(EV)
BAI3(p*tal2h*)
BAI3Δ*talI*(p*tal2h*)(EV)
BAI3Δ*talI*(p*tal2h*)(p*talI*$_{BAI3}$)
BAI3Δ*talI*(p*tal2h*)(p*talI*$_{MAI1}$)
BAI3Δ*talI*(p*tal2h*)(p*talI*$_{MAI1}$ΔAD)
talB
talA
talD
talC
talF/talE
talG
talI
talH
180 kD
130 kD
70 kD
tal2h
